# Cutaneous Granular Cell Tumor with Overlying Hypertrichosis in an Adult: A Rare Case Report

**DOI:** 10.3390/dermatopathology13010011

**Published:** 2026-03-20

**Authors:** Yara Alhusaini, Abdulaziz Almufadhi, Naif Alzahrani, Nawaf Alqahtani, Ohoud Aljarbou

**Affiliations:** 1 Department of Dermatology, King Abdulaziz Medical City, Riyadh 11426, Saudi Arabia; abdullazizalmofadhi@gmail.com; 2 College of Medicine, King Saud Bin Abdulaziz University for Health Sciences, Riyadh 11481, Saudi Arabia; alzahranin4if@gmail.com; 3 Department of Dermatology, King Saud Bin Abdulaziz University for Health Sciences, Riyadh 11481, Saudi Arabia; alqahtanin148@gmail.com; 4 Department of Pathology, King Abdulaziz Medical City, Riyadh 11426, Saudi Arabia

**Keywords:** granular cell tumor, cutaneous neoplasms, hypertrichosis, schwann cell tumor, dermatopathology, rare presentation

## Abstract

Granular cell tumors are uncommon neoplasms of neural origin that may involve the skin and often present with nonspecific clinical features, making diagnosis challenging. Cutaneous granular cell tumors rarely exhibit overlying hypertrichosis, a finding that may obscure their clinical recognition. In this report, we describe a rare case of a primary cutaneous granular cell tumor with prominent overlying terminal hair growth in an adult patient. A 27-year-old woman presented with a slowly enlarging, firm, pigmented plaque on the upper back associated with pruritus and increased hair growth. Histopathologic examination revealed sheets of large polygonal cells with abundant granular eosinophilic cytoplasm, and immunohistochemical staining was positive for S100, SOX10, CD68, and calretinin, confirming the diagnosis. The lesion was completely excised with no evidence of malignancy. To our knowledge, this represents the second reported instance of a cutaneous granular cell tumor associated with hypertrichosis and the first described in an adult. It underscores the importance of clinicopathologic correlation in evaluating unusual cutaneous lesions and expands the spectrum of recognized presentations of cutaneous granular cell tumors.

## Simple Summary

Granular cell tumors are rare neoplasms of neural origin that may involve the skin and often present with nonspecific clinical features. Accurate diagnosis typically relies on histopathologic examination. Overlying localized hypertrichosis is an uncommon finding in cutaneous granular cell tumors and may further complicate clinical assessment. In this report, we describe a primary cutaneous granular cell tumor with prominent overlying terminal hair growth in an adult patient. This presentation has been reported only once previously in the literature in a pediatric case. Recognition of this rare clinical feature is important, as skin lesions associated with localized hypertrichosis are frequently attributed to other benign or malignant conditions. This case expands the known clinical spectrum of cutaneous granular cell tumors and emphasizes the importance of clinicopathologic correlation in achieving an accurate diagnosis.

## 1. Introduction

Granular cell tumor (GCT) is an uncommon neoplasm believed to originate from Schwann cells of peripheral nerves. First described by Abrikossoff in 1926, it most frequently involves the skin, subcutaneous tissue, and oral cavity, although it has been reported in a wide range of anatomic sites. Clinically, cutaneous GCT typically presents as a solitary, slow-growing, firm nodule and is most often diagnosed in adults. Histopathologically, the tumor is characterized by large polygonal cells with abundant eosinophilic granular cytoplasm and small uniform nuclei, reflecting the presence of numerous cytoplasmic lysosomes. Immunohistochemical staining usually demonstrates positivity for S100 protein and other neural-associated markers, supporting Schwann cell differentiation.

Although most granular cell tumors follow a benign course, malignant transformation has been reported in approximately 1–2% of cases. Several unusual clinical presentations of cutaneous GCT have been described, including lesions associated with localized hypertrichosis. The mechanism underlying this phenomenon remains poorly understood but may involve local neurotrophic signaling or tumor-related stimulation of hair follicle activity.

Reports of granular cell tumors with overlying hypertrichosis are rare in the literature. Recognition of this presentation is important because the clinical appearance may mimic other cutaneous tumors that are more commonly associated with excessive hair growth. In this report, we describe a rare case of a cutaneous granular cell tumor with prominent overlying hypertrichosis in an adult patient and discuss the clinical and histopathologic features of this unusual presentation.

## 2. Case History

**Figure 1:**
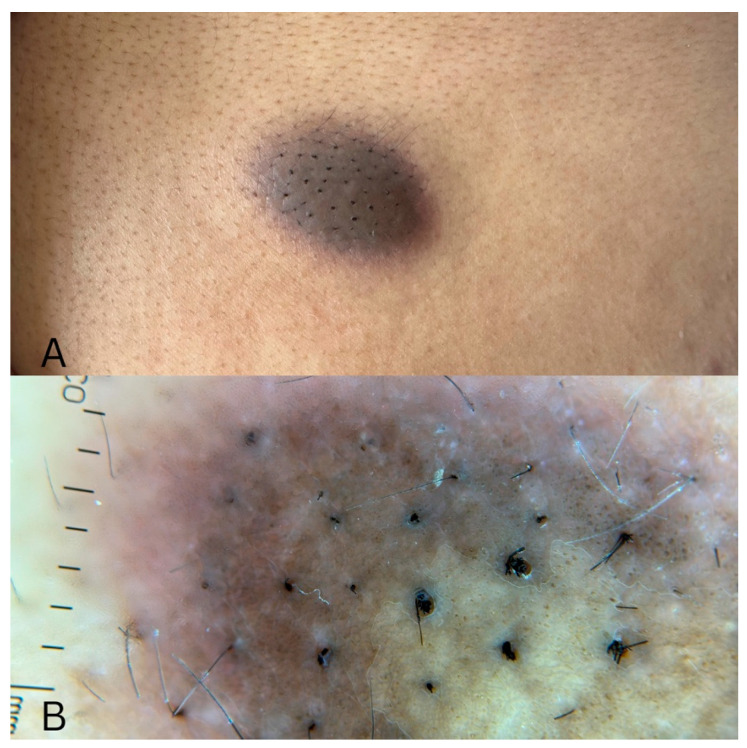
(**A**) Well-defined brown to violaceous plaque measuring approximately 2 × 2 cm on the upper back, showing prominent overlying terminal hair growth. (**B**) Dermoscopy revealed a subtle light-brown pigmented network with patchy globules and prominent terminal hairs emerging from the lesion.

A 27-year-old previously healthy female presented to the dermatology clinic with a gradually enlarging lesion on her back. She reported initially noticing a flat brown papule two years earlier, which slowly increased in size. The lesion was associated with intermittent pruritus and occasional pain, particularly after scratching. There was no history of trauma, prior similar lesions, or systemic symptoms. Cutaneous examination revealed a well-defined, 2 × 2 cm brown to violaceous plaque on the upper back with prominent overlying terminal hair growth (Figure 1A). The lesion was firm and non-tender on palpation. Dermoscopy examination revealed a subtle light brown pigmented network with patchy globules and prominent terminal hairs (Figure 1B).

Based on the clinical appearance, the differential diagnoses included dermatofibroma, dermatofibrosarcoma protuberans (DFSP), congenital melanocytic nevus, Spitz nevus, and mastocytoma.

**Figure 2:**
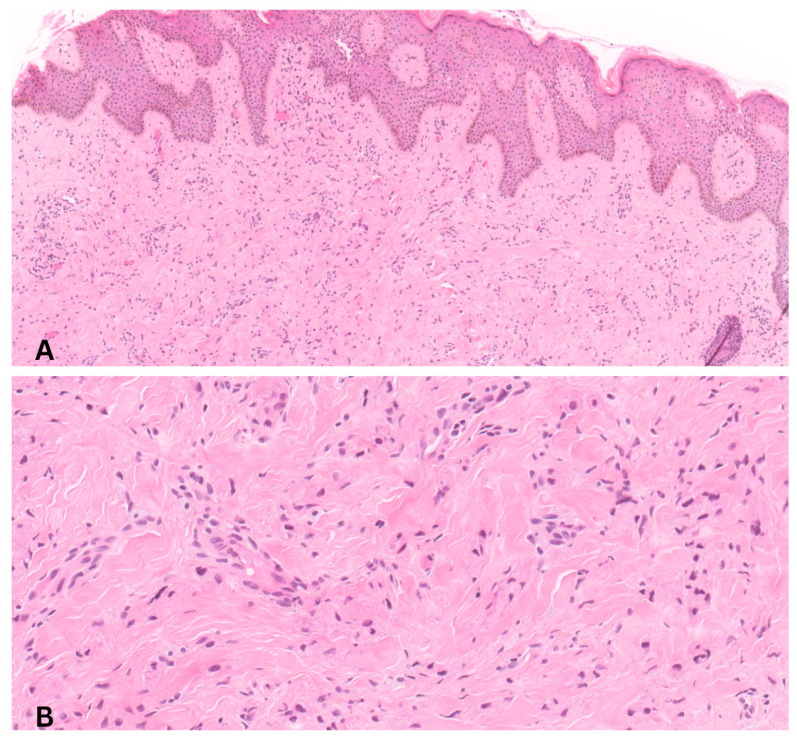
(**A**) Low-power hematoxylin and eosin (H&E) stain showing a poorly circumscribed dermal proliferation of large polygonal cells infiltrating the dermis (original magnification ×100). (**B**) High-power hematoxylin and eosin (H&E) section demonstrating a poorly circumscribed dermal proliferation composed of polygonal cells with eosinophilic cytoplasm infiltrating between collagen bundles (original magnification ×400). Hair follicles were not present in the examined histologic sections; therefore, a direct histologic correlation between the tumor and follicular structures could not be demonstrated.

A punch biopsy was performed. Histologic sections demonstrated an unremarkable epidermis with a poorly demarcated dermal lesion composed of sheets of large epithelioid to polygonal cells with abundant granular eosinophilic cytoplasm (Figure 2). Immunohistochemical staining showed strong positivity for S100, CD68, SOX10, and calretinin, with negative staining for CD34. These findings were consistent with a granular cell tumor.

Given the diagnosis and the small but recognized risk of malignant transformation (approximately 1–2% of cases), the patient was referred to plastic surgery, where the lesion was surgically excised with primary closure. Histopathologic examination of the excised specimen confirmed a benign granular cell tumor. The postoperative course was uneventful. The patient was reassured regarding the benign nature of most granular cell tumors and the importance of complete excision to minimize the risk of recurrence. At 3-month follow-up, no evidence of local recurrence was observed.

**Figure 3:**
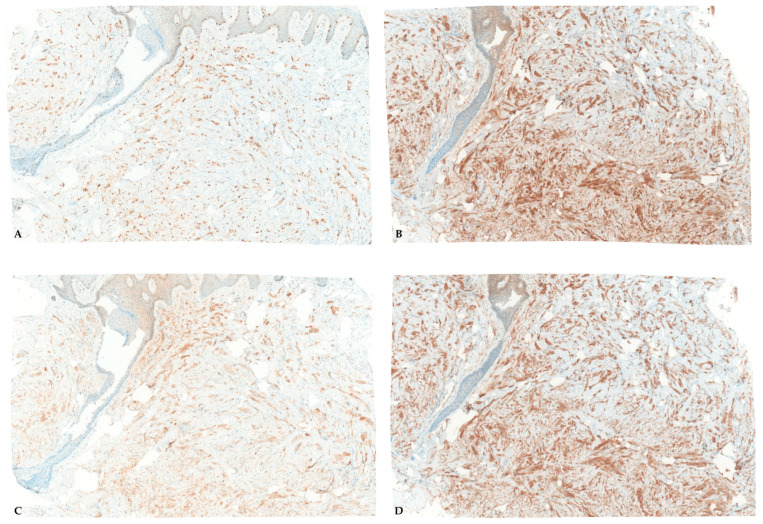
Immunohistochemical staining demonstrates diffuse tumor cell positivity for (**A**) SOX10 and (**B**) S100 protein, confirming neural crest differentiation and Schwann cell lineage. Tumor cells also show cytoplasmic positivity for (**C**) calretinin and (**D**) CD68, which highlights the lysosome-rich granular cytoplasm characteristic of granular cell tumors rather than true histiocytic differentiation. (**A**,**C**) Original magnification ×100; (**B**,**D**) original magnification ×400.

Immunohistochemical studies demonstrated diffuse nuclear positivity for SOX10 (Figure 3A), supporting Schwann cell lineage and aiding in the distinction from histologic mimickers. The tumor cells also showed diffuse and strong nuclear and cytoplasmic positivity for S100 protein (Figure 3B), confirming neural crest differentiation. In addition, the cells expressed calretinin (Figure 3C), which has been reported in a subset of granular cell tumors and further supports neural differentiation. Finally, CD68 (Figure 3D) immunoreactivity highlighted the lysosome-rich granular cytoplasm of the tumor cells and was interpreted as a cytoplasmic staining pattern rather than evidence of histiocytic differentiation.

## 3. Discussion

Granular cell tumors (GCTs) are uncommon neoplasms of neural origin that may arise in a wide range of anatomic locations, most frequently involving the head and neck region. Primary cutaneous involvement remains relatively rare and often presents a diagnostic challenge because of the nonspecific clinical morphology of these lesions [1,2]. Cutaneous GCTs typically manifest as slow-growing, firm nodules or plaques with variable pigmentation and minimal surface change, frequently mimicking a broad spectrum of benign and malignant dermatologic conditions. Consequently, clinical diagnosis alone is rarely definitive, and histopathologic examination remains essential for accurate diagnosis.

The present case is notable for the presence of prominent localized hypertrichosis overlying the tumor. To date, this feature has been reported only once previously in the literature in a pediatric patient described as a “hairy” granular cell tumor [3]. In contrast, our case occurred in an adult patient and demonstrated similar terminal hair growth despite differences in patient age and anatomic location. These findings suggest that hypertrichosis may represent an intrinsic biologic characteristic of certain granular cell tumors rather than an age-dependent phenomenon.

The pathophysiologic basis of hypertrichosis in association with GCT remains incompletely understood. Given the Schwann cell derivation of granular cells, one plausible explanation involves tumor-related modulation of perifollicular neural signaling. Peripheral nerves play an important role in regulating hair follicle cycling through neuropeptides and growth factors that influence follicular stem cell activity. Similar mechanisms have been proposed in other neural and neuromuscular lesions associated with localized hypertrichosis, including plexiform neurofibromas and smooth muscle hamartomas [4,5]. Local production of neurotrophic factors or altered neural–epithelial interactions may therefore promote terminal hair growth overlying the lesion.

The occurrence of localized hypertrichosis over cutaneous tumors is uncommon but has been reported in association with several neural or mesenchymal lesions. In the present case, the lesion demonstrated prominent localized hypertrichosis clinically. The clinical appearance also raises the possibility of polytrichia, defined as the presence of multiple hair shafts emerging from a single follicular opening. Although this feature was not definitively confirmed histologically, the dense terminal hair growth observed over the lesion may reflect follicular stimulation induced by tumor-derived neurotrophic factors or local microenvironmental changes.

From a dermatopathologic perspective, granular cell tumors show distinctive morphologic and immunophenotypic features that allow reliable diagnosis when adequately sampled. However, diagnostic pitfalls remain well recognized; for instance, secondary pseudoepitheliomatous hyperplasia of the overlying epidermis may simulate well-differentiated squamous cell carcinoma. Awareness of this phenomenon is critical to avoid misinterpretation and unnecessary aggressive treatment. In addition, the granular cytoplasm of tumor cells may lead to confusion with other granular cell-containing lesions, emphasizing the importance of clinical awareness and immunohistochemical correlation [6].

Granular cell tumors may demonstrate a broad range of clinicopathologic presentations depending on their anatomic location and depth of involvement. While the majority of lesions arise within the dermis or subcutaneous tissue, involvement of mucosal surfaces, internal organs, and peripheral nerves has also been documented. Cutaneous lesions may exhibit variable pigmentation, induration, or plaque-like morphology, which can lead to diagnostic confusion with dermatofibroma, dermatofibrosarcoma protuberans, or melanocytic lesions. In such cases, histopathologic evaluation remains the cornerstone of diagnosis, utilizing the established *WHO Classification of Skin Tumors* to ensure diagnostic precision.

Most cutaneous granular cell tumors follow an indolent clinical course and enlarge slowly over time, which may delay patient presentation. Although the vast majority of GCTs are benign, malignant variants have been described and are estimated to account for approximately 1–2% of cases [7,8]. Clinical features that may raise suspicion for malignancy include rapid enlargement, ulceration, local invasion, or recurrence. For this reason, complete surgical excision with histologically clear margins remains the treatment of choice, permitting definitive diagnosis while minimizing the risk of recurrence [9].

Granular cell tumors should therefore be considered in the differential diagnosis of indurated or pigmented cutaneous lesions with unusual clinical features. Immunohistochemistry plays a critical role in confirming neural differentiation, with diffuse positivity for S100 protein and SOX10 supporting Schwann cell origin [10]. Recognition of the characteristic granular cytoplasm allows distinction of granular cell tumors from other entities that may exhibit granular cell change. Awareness of these clinicopathologic correlations is essential for dermatologists when evaluating atypical lesions, as accurate diagnosis can prevent unnecessary delay in treatment [11].

The current case expands the recognized clinical spectrum of cutaneous granular cell tumors by documenting localized hypertrichosis in an adult patient. Recognition of this unusual presentation is important for both clinicians and dermatopathologists, as hypertrichotic lesions may initially suggest alternative diagnoses. Early biopsy, accurate clinicopathologic correlation, and appropriate surgical management are essential to ensure optimal patient outcomes and reassurance. Continued reporting of similar cases may further clarify the biological relationship between neural tumors and localized hypertrichosis [12].

## 4. Conclusions

Cutaneous granular cell tumors are uncommon neoplasms that may present with nonspecific clinical features, frequently posing a diagnostic challenge for clinicians. The present case highlights a rare manifestation characterized by prominent localized hypertrichosis overlying the lesion. Recognition of this unusual presentation is important because hypertrichotic cutaneous lesions may clinically mimic a variety of benign or malignant dermatologic conditions. Histopathologic examination combined with immunohistochemical analysis remains essential for establishing the diagnosis and distinguishing granular cell tumors from other lesions demonstrating granular cell change. Complete surgical excision remains the treatment of choice and is associated with an excellent prognosis in benign cases. By documenting localized hypertrichosis in an adult patient, this report expands the clinical spectrum of cutaneous granular cell tumors and underscores the importance of clinicopathologic correlation when evaluating atypical hypertrichotic skin lesions. Continued reporting of similar cases may further clarify the biologic relationship between neural tumors and localized alterations in hair follicle growth.

## Data Availability

The data presented in this study are available within the article.

## References

[1] Fahim S., Aryanian Z., Ebrahimi Z., Kamyab-Hesari K., Mahmoudi H., Alizadeh N., Heidari N., Livani F., Ghanadan A., Goodarzi A. (2022). Cutaneous granular cell tumor: A case series, review, and update. J. Fam. Med. Prim. Care.

[2] Montazer F., Kazeminejad A., Rokni G.R., Tayebi S. (2018). Case report: Cutaneous granular cell tumors. F1000Research.

[3] Liu T., Han Y., Zheng S., Li B., Liu Y., Chen Y., Liu Y., Wang E. (2015). Primary cutaneous malignant granular cell tumor: A case report and literature review. Diagn. Pathol..

[4] Palo S., Mishra M., Mulsange K.A. (2025). Cutaneous Granular Cell Tumor in a Middle-aged Woman: Clinicopathological Insights from a Rare Schwannian Neoplasm. J. Midlife Health.

[5] Cambiaghi S., Maffeis L., Boneschi V. (2010). “Hairy” granular cell tumor. Pediatr. Dermatol..

[6] Amphlett A. (2022). An update on cutaneous granular cell tumours for dermatologists and dermatopathologists. Clin. Exp. Dermatol..

[7] Pérez-González Y.C., Pagura L., Llamas-Velasco M., Cortes-Lambea L., Kutzner H., Requena L. (2015). Primary cutaneous malignant granular cell tumor: An immunohistochemical study and review of the literature. Am. J. Dermatopathol..

[8] Fanburg-Smith J.C., Meis-Kindblom J.M., Fante R., Kindblom L.G. (1998). Malignant granular cell tumor of soft tissue: Diagnostic criteria and clinicopathologic correlation. Am. J. Surg. Pathol..

[9] LeBoit P.E., Burg G., Weedon D., Sarasin A. (2006). World Health Organization Classification of Tumours of the Skin.

[10] Mukhopadhyay S., Katzenstein A.L. (2011). Subcutaneous granular cell tumor: Evidence supporting Schwann cell origin. Arch. Pathol. Lab. Med..

[11] Rose B., Tamvakopoulos G.S., Yeung E., Pollock R., Skinner J., Briggs T., Cannon S. (2009). Granular cell tumours: A rare entity in the musculoskeletal system. Sarcoma.

[12] Tronnier M., Wolff H.H. (1995). Localized hypertrichosis associated with underlying tumors. J. Am. Acad. Dermatol..

